# Role of Mesenchymal Stromal Cells as Therapeutic Agents: Potential Mechanisms of Action and Implications in Their Clinical Use

**DOI:** 10.3390/jcm9020445

**Published:** 2020-02-06

**Authors:** Gonzalo José Jimenez-Puerta, Juan Antonio Marchal, Elena López-Ruiz, Patricia Gálvez-Martín

**Affiliations:** 1 Biosanitary Research Institute of Granada (ibs.GRANADA), University Hospitals of Granada-University of Granada, 18016 Granada, Spain; gonzalojimenez@telefonica.net (G.J.J.-P.); jmarchal@go.ugr.es (J.A.M.); 2 Biopathology and Regenerative Medicine Institute (IBIMER), Centre for Biomedical Research (CIBM), University of Granada, 18016 Granada, Spain; 3 Department of Human Anatomy and Embryology, Faculty of Medicine, University of Granada, 18016 Granada, Spain; 4 Excellence Research Unit “Modeling Nature” (MNat), University of Granada, 18016 Granada, Spain; 5 Department of Health Sciences, University of Jaén, 23071 Jaén, Spain; 6 Department of Pharmacy and Pharmaceutical Technology, Faculty of Pharmacy, University of Granada, 18016 Granada, Spain; 7 R&D Human Health, Bioibérica S.A.U., 08029 Barcelona, Spain

**Keywords:** mesenchymal stromal cells, mechanism of action, homing, immunomodulation

## Abstract

Due to the great therapeutic interest that involves the translation of mesenchymal stromal cells (MSCs) into clinical practice, they have been widely studied as innovative drugs, in order to treat multiple pathologies. MSC-based cell therapy involves the administration of MSCs either locally or systemically into the receptor body where they can traffic and migrate towards the affected tissue and participate in the process of healing. The therapeutic effects of MSCs compromise of different mechanisms such as the functional integration of differentiated MSCs into diseased host tissue after transplantation, their paracrine support, and their impact on the regulation of both the innate and the acquired immune system. Here, we establish and provide recent advances about the principal mechanisms of action through which MSCs can perform their activity and effect as a therapeutic tool. The purpose of this review is to examine and discuss the MSCs capacity of migration, their paracrine effect, as well as MSC-mediated modifications on immune cell responses.

## 1. Introduction

Mesenchymal stromal cells (MSCs) are multipotent cells which are recognized for being a subset of non-hematopoietic adult stem cells originating from the mesoderm layer, with fibroblast-like morphology and multipotent potential [1,2]. MSCs are capable of differentiating into mesodermal lineages, such as adipocytes, osteocytes, or chondrocytes, and also into endodermic and neuroectodermic lineages, such as alveolar endothelial cells or neurons [3,4,5].

Moreover, they are self-renewable and culturally expandable in vitro with few ethical issues, marking their importance in cell therapy and tissue repairment. MSCs were first isolated from bone marrow by Friedenstein et al. in the 1960–1970s [6,7]. However, presently it is known that MSCs exist in almost all tissues. They have been isolated from various human sources, such as the umbilical cord, umbilical cord blood, adipose tissue, amniotic fluid, peripheral blood, muscle, and many organs including fetal liver, brain, lung and so on [4,8].

Although MSCs were successfully derived from all of these tissues, there are practical limitations such as the difficulty and invasiveness of the procurement [9]. Moreover, MSCs from different tissues exhibit varied in vitro characteristics, including their proliferation capacity and differentiation potential, which influence their applicability [10,11,12,13,14,15]. Therefore, selection of an adequate cell source for their clinical use should ideally be based on their logistical, practical, and functional behavior [10]. Table 1 describes the advantages and disadvantages of MSCs from the three main sources that have been investigated in clinical studies: bone marrow, adipose tissue, and the umbilical cord [2] (Table 1).

In order to clarify and harmonize what the fundamental charactericts of MSCs are, the International Society for Cellular Therapy (ISCT) proposed three minimal criteria for cultured human MSCs definition: (i) MSCs must be plastic-adherent; (ii) MSCs must have trilineage differentiation potential in vitro into osteoblasts, adipocytes, and chondroblasts; and (iii) MSCs must be positive (>95%) and negative (<2%) for a panel of cell surface antigens. Human MSC marker expression must include positive markers, such as CD105, CD73, and CD90, and negative markers such as CD34, CD45, CD79 or CD19, CD14 or D11b, and HLA-DR [16]. However, the panel of MSC markers is growing rapidly and promising markers—which could reach a better MSC identification and enrichment of the stem cell population—such as CD271 (low affinity nerve growth factor Receptor [LNGFR]), stage-specific embryonic antigen 4 (SSEA-4), or stromal cell antigen 1 (Stro-1) have been proposed [8].

MSCs can migrate to the accurate place of injury [17], where they can differentiate and replace damaged resident cells and promote tissue regeneration, as demonstrated in preclinical models of heart [18], pancreas [19], kidney [20], and liver [21]. However, MSCs not only provide healing capacities performed through engraftment and differentiation, but also through paracrine signaling and communication through cell-cell contact. These cells are capable of secreting soluble factors, which are indispensable for cell viability and proliferation, and for modulating the immune response.

MSC-based cell therapy involves the administration of MSCs into the receptor body, migrating (or ‘’homing’’) towards the affected tissue, and causing direct and indirect modifications over the immune system and microenvironments acting as a therapeutic agent [22]. MSCs migrate to the injury site, in response to chemotactic signals released in response of tissue damage [23]. Moreover, the effects of MSCs are partly based on their capacity to immunomodulate their environment. They are involved in immunoregulation by interacting with both myeloid and lymphoid cells of innate and adaptive immune systems, making no-immune activation [24,25]. Among the therapeutic possibilities of MSC-immune interactions, evidence greatly supports a remarkable effect as immunomodulators through the secretion of soluble factors and the release of extracellular vesicles (EVs) [26].

Due to the great therapeutic interest involving the clinical translation of MSCs, they are being widely studied to determine their mechanism of action (MoA) in order to evaluate their efficacy, feasibility, and safety in humans to treatment of numeorus pathologies [27]. Based on their capacity to treat or improve the function of a damaged tissue or organs in patients, MSCs are considerated medicines from the regulatory point of view [28]. Thus, according to each jurisdiction the MSC-based products are regulated under a specific regulatory framework. In Europe, the European Medicine Agency classifies human MSCs-based products as Advanced Therapies Medicinal Products (ATMPs). The ATMPs are regulated under the Regulation No. 1394/2007. On the other hand, in United States of America, human MSCs are classified by the Food and Drug Administration (FDA) as Human Cellular and Tissue-based Products (HCT/Ps), and are regulated as a biologic drug under Code of Federal Regulations, part 1271(21 CFR 1271) [29].

This work reviews the latest advances in MSC-based cell therapy in order to update the results about the main MoA of MSCs as therapeutic agents for their clinical application. In particular, we focus on three functions and mechanisms for applying this knowledge to get maximum repair and regeneration of cell-derived therapeutics for regenerative medicine. Then, we focus on MSCs capacity of migration, their paracrine effect, as well as MSC-mediated modifications on immune cell responses.

## 2. MSCs Mechanisms of Action

As MSCs perform their activity over the damaged tissue, it is necessary to know the potential mechanisms that may be involved in their therapeutic action. In the last few years, several reports have been published about the crucial MoA through which MSCs perform their role as keepers of tissue homeostasis, as well as a tool for regenerative medicine.

Despite the evidence about MSCs direct differentiation and cell replacement, recent studies strongly suggest that the most remarkable MoA of MSCs are attributed to their capacity of migration [17] and paracrine effect [30,31], as well as the modifications that MSCs can cause over the immune system (immunomodulation) [32,33].

### 2.1. Homing and Migration of MSCs

MSCs homing depends on their source of origin, that is, whether they are exogenous (injected) or they simply act endogenously from one of its body reservoirs. MSCs can be exogenously administered either locally or systemically. The local administration implies a non-vasculatory process where MSCs are allocated at the target tissue [34].

**Figure 1:**
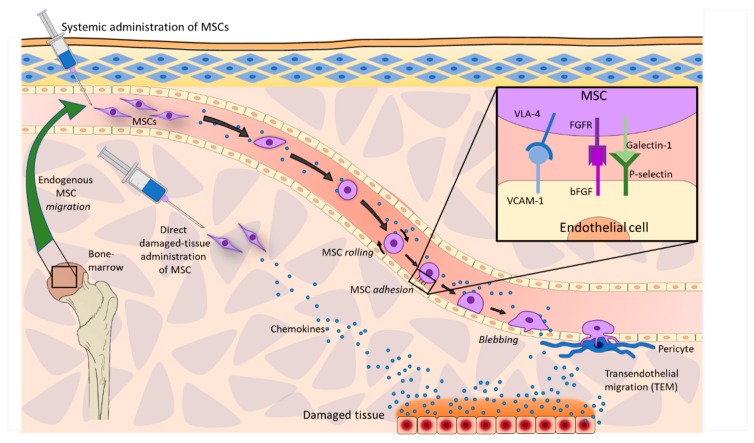
Homing and transendothelial migration of mesenchymal stromal cells (MSCs). MSCs can act both endogenously or exogenously, performing a homing process, by which they move towards the affected site following chemotactic signals in order to repair or contribute to the recovery of the damaged tissue. bFBF: basic fibroblast growth factor; FGFR: fibroblast growth factor receptors; VLA-4: very late antigen 4; VCAM-1: vascular cell adhesion molecule 1.

On the other hand, the process of systemic MSC homing requires three phases:(i) direct administration into blood vessels, (ii) extravasation at the lesion vicinity, and (iii) interstitial migration toward the target site [23,34]. The extravasation and interstitial migration are commonly arranged to the term “transendothelial migration” (Figure 1).

Independently of their origin, MSCs tend to enter into the vascular system in order to easily reach the affected site. However, MSCs exhibit a remarkable tendency to get located in non-specific tissues, such as the microvasculature and the capillary network, regardless of the presence or absence of an specific injury and the multiple surface leukocyte-like receptors, which intervene in MSC homing [23,35]. MSCs must approach the damaged site through the vascular system and then pass through the endothelial wall in order to keep moving towards the lesion [17].

MSC extravasation is initiated by proinflammatory chemokines from the injury site, as tumor necrosis factor-alpha (TNF-α) or histamine, which can activate endothelial cells of blood vessels, and thus, induce P-selectin activation as well as vascular cell adhesion molecule 1 (VCAM-1) and the expression of the intercellular adhesion molecule 1 (ICAM-1) on the endoluminal surface [36]. Then, flowing MSCs begin to roll over the apical endothelial surface and the endothelium starts to upregulate local ligands for extravasation as CD44 (homing cell adhesion molecule (HCAM)), CD49d, and very late antigen 4 (VLA-4) [37]. When MSCs adhere to endothelium ligands, a signal expression pattern is released ensuring a firm adhesion [23] (Figure 1). Extravasation involves, at first, the development of a contact surface on the MSCs to be able to adhere to endothelial cells.

MSC tethering is dependent on the molecule adhesion pattern on its surface, which is formed by galectin-1 and a huge variety of integrins, such as VLA-4. These molecules interact with P-selectin and VCAM-1 in the endothelium surface, respectively. Besides, MSCs express platelet and neutrophil derived molecules such as fibroblast growth factor receptors (FGFR), which interact with basic fibroblast growth factor (bFBF) of endothelial cells and mediate in the adhesion of galectin-1 to P-selectin [23,38].

The molecular process starts with the recognition of proinflammatory cytokines, such as interleukin-8 (IL-8), which activates phospholipase C (PLC), resulting in an increase at the inner calcium (Ca2+) levels [23,39]. The rising levels of Ca2+ trigger a downstream signaling in which guanine-exchange factors (GEF), GTPases (Rho and Rap1) and, tailin (an intracellular adapter protein) are implied [23,40]. VLA-4 leads to a straightened position, on which the binding pocket is exposed and so conferring affinity to the integrin. Higher affinity can be achieved by a PLC-Rho/Rap1-independent way, which involves phosphokinase C (PKC), phosphoinositide 3-kinase (PI3K)/Act, and other adapter proteins such as integrin-linked kinase (ILK) [23].

**Figure 2:**
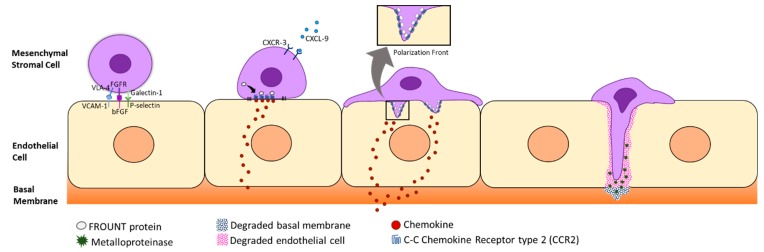
Transendothelial migration through the endothelial cells of the endothelium. Mesenchymal stromal cells (MSCs) develop a front pole through the joint action of the FROUNT protein and the C-C chemokine receptor type 2 (CCR2) in order to degrade endothelial cells and its basal membrane on its way to the damaged site.

Once MSCs are attached to the endothelium, they form filopodia thanks to chemokine ligand 9 (CXCL-9) stimulation [41], allowing MSCs to cross the intraluminal space by using proteases such as metalloproteases. At the same time, MSCs get polarized developing a front pole by the action of an intracellular adaptor protein called FROUNT, which is linked to the C-C Chemokine Receptor type 2 (CCR2). When CCR2 starts to cluster, it results in a cytoskeletal reorganization, which includes enhanced actin polymerization [13]. Other C-C chemokine receptors (apart from CCR2) are expressed by MSCs, such as CCR1, CCR4, CCR7, CCR9, CCR10, CXCR1, CXCR3, CXCR5, CXCR6, CX3CR1, and CXCR4, which are thought to perform a role in MSC homing. However, the predominant chemokines in MSC homing remain unknown [42,43,44] (Figure 2).

MSCs overcome the endothelial basement membrane by the interaction of different extracellular metalloproteases [45,46], which degrade the basal membrane type IV collagen [23,47]. When MSCs have approached the damaged site through the vascular system, they extravasate in order to keep moving towards the lesion. MSC extravasation has been reported to be essential for MSCs in order to perform their regenerative and restoring capacities effectively [48]. The migration finishes once MSCs have reached their target, where they are expected to perform their therapeutic activity in order to restore functionality [49].

### 2.2. MSCs and Immune System

The response of MSCs after a stimulus is conditioned by the signals that these cells receive from their surrounding microenvironment [50], such as inflammatory cytokines [51] or hypoxia factors [52]. After a stimulus, MSCs secrete immunoregulatory factors which eventually suppress or enhance the immune response based on the physiological context [49,53].

**Figure 3:**
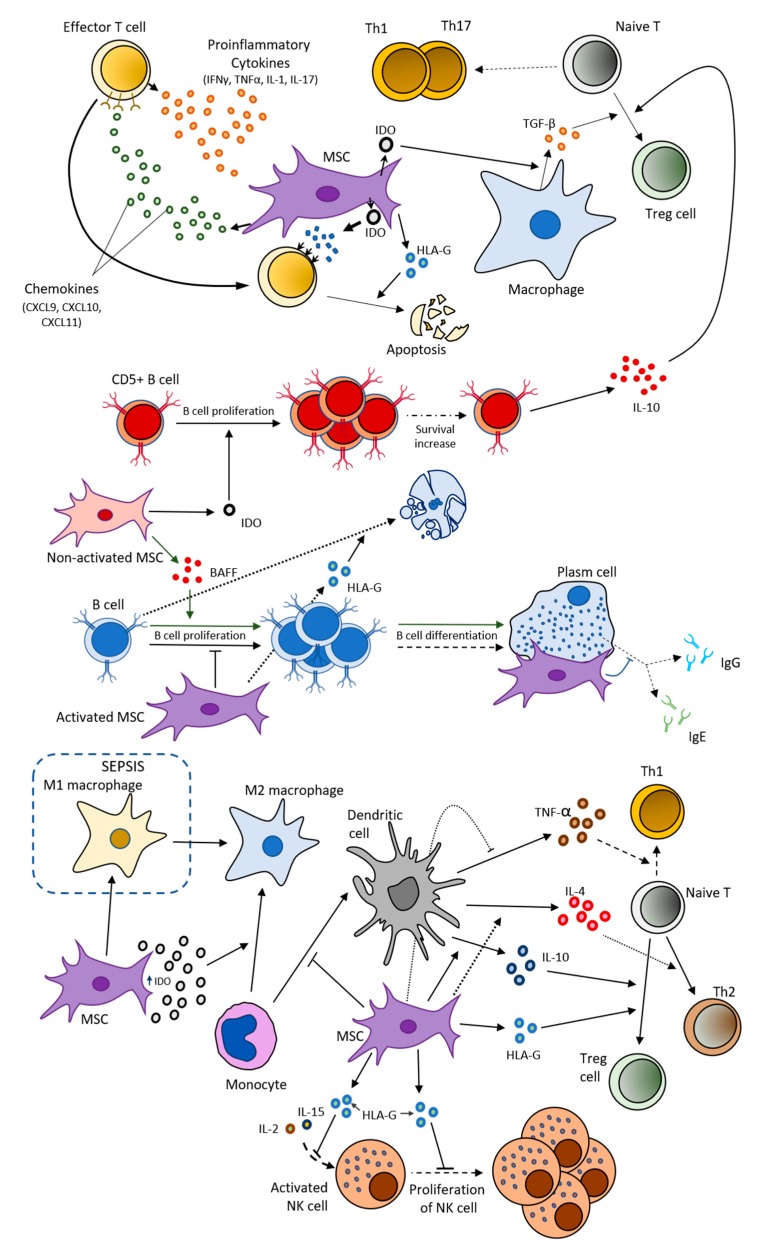
Immune interactions (immunomodulation) of mesenchymal stromal cells (MSCs). MSCs have been proven to have influence over both adaptative cells, such as T and B cells, and innate immune cells, such as dendritic cells, natural killer (NK) cells, monocytes, and macrophages by secreting several molecular factors as indoleamine 2,3-dioxygenase (IDO) and different cytokines. BAFF: B cell activating factor.

The production of these factors by MSCs also relies upon their phenotype, which comes as a result of the interaction between the paracrine factors from their surrounding microenvironment with the receptors presented on MSCs surface, such as Toll-like receptors (TLR) [54]. According to Waterman et al., two different MSC phenotypes can be distinguished: proinflammatory MSC phenotype (MSC1; accomplished by TLR4 interaction with its ligand) and anti-inflammatory MSC phenotype (MSC2; as a result of TLR3 bounding with its ligand) [50]. Both MSC1 and MSC2 are recognized to produce two specific types of immunodulator factors, proinflammatory and anti-inflammatory factors (also known as immunosuppressive factors), respectively [50] (Figure 3).

The status of pro-inflammatory or immunosuppressive is determined by the effects they can cause over the immune cells. Thus, MSCs can ‘’immunomodulate’’ the activation and function of several immune cells, both innate and adaptive cells, like T lymphocytes, macrophages, natural killer, B lymphocytes, neutrophils, and dendritic cells [49]. MSCs are essential for T cell survival. In acute inflammatory phases, effector T cells start to secrete a great number of proinflammatory cytokines (IFN-γ, TNF, IL-1, and IL-17) which activates MSCs [30]. Then, MSCs start their immunomodulation by releasing large amounts of prostaglandin E2 (PGE2), IL-10, Human Leukocyte Antigen-G (HLA-G), indoleamine 2,3-dioxygenase (IDO), and chemokines, as CXCL9, CXCL10, and CXCL11 (ligands of the T cell-specific chemokine receptor, CXCR3 [30,55]. Likewise MSCs secrete CXCL12 (or stromal cell-derived factor 1 alpha [SDF-1α]) and CXCR4 [56]. These chemokines attract T cells to the close proximity of activated MSCs, where IDO is catabolized and its catabolites inhibit T cells, eventually resulting in T cell apoptosis.

Moreover, T cell proliferation and their differentiation into helper T cells, like Th1 and Th17, can be inhibited by MSC secretome which includes HLA-G, galectins, and IDO among other molecules, enabling macrophages to produce transforming growth factor-β (TGF-β) and promoting T regulatory cells (Treg) differentiation [55,57,58]. Indeed, HLA-G can promote the apoptosis of both T and B lymphocytes, impair the cytolysis of antigen-activated CD8+ cells, promote the activation of CD4+CD25+ FoxP3+ regulatory T cells, and disturb the cytolysis, adhesion, and migration capacities of natural killer (NK) cells. Therefore, HLA-G acts by decreasing the excessive immune response of autoimmune disorders [55,59] (Figure 3).

Other molecules produced by MSCs, known as galectins, are crucial in their immunosuppressive effect. MSC-derived galectin-1 significantly disrupts the release of inflammatory cytokines, such as TNFα, IFNγ, IL-2, and IL-10. Galectin-3 regulates the T cell proliferation, adhesion, and migration. In addition, galectin-3 mediates in the reduction of stimulated T and B lymphocytes alongside galectin-1 and galectin-9 [60,61].

Interestingly, the production of IDO is related to anti-inflammatory cytokine levels, as TGF-β, which means that decreased levels of TGF-β match to diminished levels of IDO, desactivating MSCs effect over immune cells and promoting immune response [62]. Therefore IDO is recognized as an on–off switch system in immunomodulation, since it determines MSCs plasticity over T cells [57].

MSCs can also perform their immunomodulative action over other adaptative immune cells, such as B cells. Activation of MSCs have been reported to inhibit B cell proliferation, plasm cell differentiation, as well as IgE and IgG releasing from activated B cells by a direct cell–cell interaction, while unstimulated MSCs do not suppress B cell proliferation and may even promote proliferation to some extent [63,64] (Figure 3).

On the other hand, adipogenic-differentiated MSCs can promote the proliferation of activated B cells by the secretion of the B cell activating factor (BAFF), while the opposite effect is observed in MSCs [65] (Figure 3). Moreover, MSCs with secreted-IDO accelerates survival and proliferation of CD5+ B cells. These cells are considered a particular B cell subpopulation with a notable immunoregulation ability performed by secreting IL-10 or inducing the differentiation of Tregs [66].

MSCs are not only associated with adaptative immune cells, but also with innate immune cells. Concretely, activated NK cells can be recognized by MSCs, and consequently, MSCs are capable of inhibiting NK cell proliferation by downregulating their activation via IL-2 or IL-15 [67]. Dendritic cells (DC) are also affected by MSCs, thus MSCs inhibit CD14+ monocyte differentiation towards DC. Besides MSCs block TNFα secretion by DC as promoting IL-10 and IL-4 secretion, which impedes T cell differentiation into Th1 cells, directing these cells to differentiate into Treg and Th2 cells, respectively [68].

Macrophages also interactact with MSCs. It has been reported that IDO high expression, as a result of proinflammatory cytokines interaction, leads monocytes to differentiate into immunosuppressive and anti-inflammatory type 2 macrophage phenotype (M2) [69]. Abumaree et al. revealed that this MSC-dependent macrophage phenotype switching could occur from a proinflammatory type 1 macrophage phenotype (M1) to an anti-inflammatory type 2 phenotype (M2) in sepsis treatment [70].

Moreover, MSCs exhibit antimicrobial activity through the secretion of antimicrobial peptides (AMPs), such as cathelicidin peptide LL-37, hepcidin, β-defensin 2, lipocalin 2, and Hepcidin [71,72].These AMPs act in destroying bacterias by either altering the integrity of the microbial membrane or by encouraging the release of proinflammatory cytokines which in turn favors the recruitment of immune cells [71]. Therefore, there is an over-stimulation of the immune cells, as the formation of neutrophil extracellular traps (NETs) by neutrophils [71].

Antimicrobial activity of MSCs can be considered counterproductive sometimes. Thus, MSCs can support the host by immunosuppressing the environment by: (i) refrying the intensification of pathological symptoms; (ii) helping to cure damaged tissue or organ; and (iii) allowing the formation of an immune tolerant environment. However, excessive immune suppression can cause the completely different effect, impeding the host from attacking the infection, and instead, favoring microbial effectors to spread [72].

### 2.3. Paracrine Activity

Several studies have shown that only a small proportion of transplanted MSCs survives and integrate into host tissues [48]. Therefore, the therapeutic effects of MSCs appear to not only depend on direct cell-to-cell interaction. MSCs are also capable of providing beneficial effects such as cell survival and proliferation, and to modulate the immune response through paracrine signaling. This paracrine activity includes the secretion of small molecules as growth factors, cytokines, chemokines, and the release of EVs that contain a wide range of molecules such as mensenger RNA, peptides/proteins, and microRNAs [73].

#### 2.3.1. Secretome

Despite the fact that the homeostatic and immunomodulatory activity of MSCs were thought to be reliant on MSC cell number, it has been seen that it depends more on the paracrine factors these cells secrete, also known as ‘’secretome’’, which is produced once MSCs are activated [49,53]. Therefore, in response to environmental cues of the injury site, MSCs are activated and secrete various cytoprotective paracrine factors to enhance regeneration of the damaged tissue [73].

**Figure 4:**
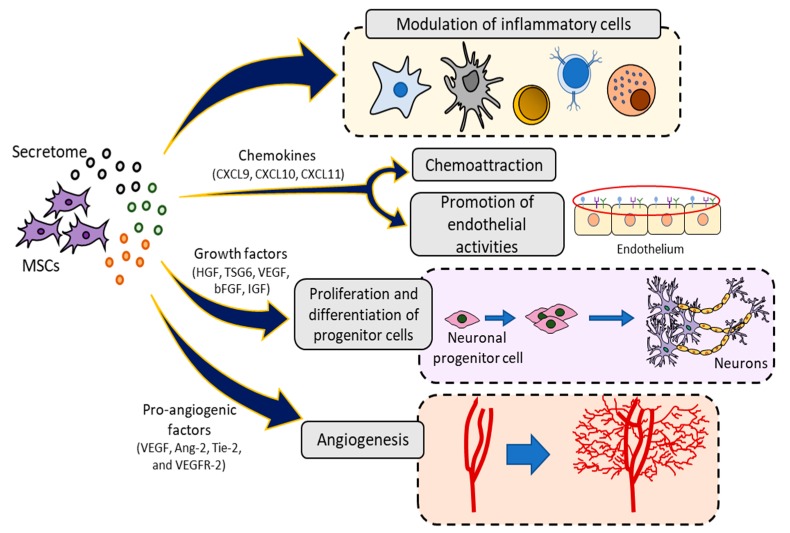
Paracrine activity of mesenchymal stromal cells (MSCs). MSC-secreted factors can interact directly at different cellular processes, such as immunomodulation, chemoattraction, progenitor cell proliferation, angiogenesis and differentiation, remaining all of them essential to the correct function of MSCs within the body. HGF: hepatocyte growth factor; IGF: insulin-like growth factor 1; TSG6: tumor necrosis factor-inducible gene 6 protein; VEGF: vascular endothelial growth factor.

Factors produced by MSCs include growth factors, such as hepatocyte growth factor (HGF), tumor necrosis factor-inducible gene 6 protein (TSG6), vascular endothelial growth factor (VEGF), bFGF, insulin-like growth factor 1 (IGF-1), chemokine (C-C motif) ligand 2 (CCL-2), epidermal growth factor (EGF), platelet-derived growth factor (PDGF), as well as IL-6, TGF-β, prostaglandin E2, IDO, and SDF-1 [4]. Together, these secreted factors may inhibit inflammatory responses, trigger angiogenic process, promote endothelial and fibroblast activities, and facilitate the proliferation and differentiation of progenitor cells into tissues in situ [49] (Figure 4).

MSC activation remains essential in the production of all these factors. Inflammatory stimuli and/or cross-talk with injured cells could enhanced the secretions and therapeutic effects of MSCs, whereas non-activated MSCs do not induce a significant increase of paracrine factors which is necessary for its therapeutic use [73]. Once MSCs are activated, the production of growth factors increases to optimum levels when they can cause an efficient response. Chemokines are released by MSCs when these cells have received any stimulus, starting the secretion of chemoattractant molecules [4].

When tissue is damaged, it suffers a fluctuant acute inflammation process. During the first phases of the acute inflammation process, proinflammatory cytokines are highly expressed and contribute to activating a larger number of MSCs leading to a better immunosuppressive response [74]. Multiple procedures have been considered to treat acute inflammation, among them, the administration of proinflammatory cytokines before MSCs, or the usage of inhibitors of anti-inflammatory cytokines previous to MSCs treatment [24].

In contrast to acute inflammation, in chronic inflammation, during remission or during immunosuppressant treatments, the production of proinflammatory cytokines diminishes as the concentration of anti-inflammatory cytokines, like TGF-β, increases. Besides, in this case, IDO production is below the immunosuppressive threshold as well as chemokines, causing T lymphocytes liberation so they can promote immune response [75,76].

In addition, immunosuppressant factors secreted by MSCs are not useful as coadjuvants to conventional immunosuppressant drugs, despite having both quite similar effects; both immunosuppressants and MSCs inhibit the inflammatory response by suppressing effector T cells. In fact, it has been observed that the immunosuppressant effect of MSCs can be reverted by the administration of immunosuppressant drugs as cyclosporine A and dexamethasone [77]. Not only can in vivo endogenous factors affect MSC migration and homing, but also both are influenced by multiple in vitro factors, concretely culture factors, such as age and passage number of the cells, the delivery method, and culture conditions among others. For instance, the higher is the number of passages, the less is the engraftment efficiency of MSCs [56].

Particularly, MSC attachment could be influenced by in vitro procedures, since the expression pattern of α4 subunit of VLA-4 has been reported to differ depending on isolation and cultivation, but also depending on individual donors and species [23].

#### 2.3.2. MSC-Derived Extracellular Vesicles

**Figure 5:**
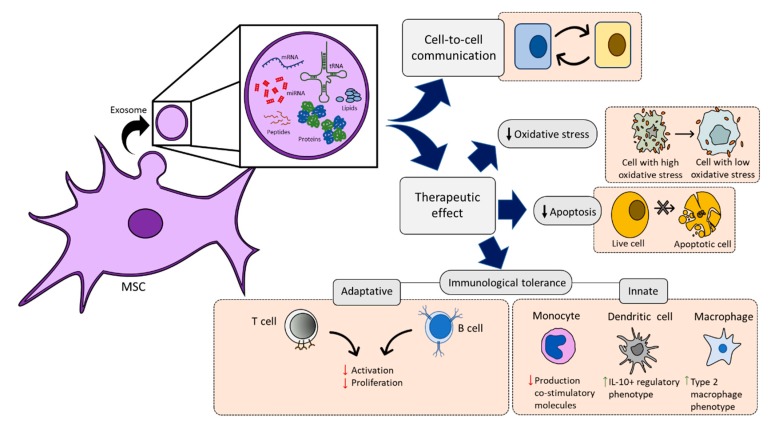
Mesenchymal stromal cell-secreted exosomes perform a role in cell-to-cell communication, as well as triggering therapeutic effects, by a reduction of oxidative stress, increasing cell live expectancy, and exhibiting an immunological tolerance through the reduction of activation and proliferation of adaptative immune cells and the differentiation of innate cells towards regulatory phenotypes.

In addition to secreted factors, the paracrine-protective mechanisms of MSCs include the production of EVs, which include exosomes, and transfer of mitochondria [32,78,79]. Recently, it has been shown that the EVs from MSCs contain vesicular cargo molecules that include messenger RNA, transfer RNA, microRNA, and peptides/proteins. In addition, the EVs enclose lipids which are able to mediate cell-to-cell communication and can exert therapeutic effects on various tissue injuries via attenuation of immune cell activation [80,81] by ameliorating oxidative stress [82] or by decreasing apoptosis [83] (Figure 5).

Therefore, exosomes are considered to play a major role in the communication between MSCs and surrounding cells. For example, it has been shown that during in vitro culture with peripheral blood mononuclear cells, MSC-derived exosomes extracted from bone marrow are able to suppress the secretion of the pro-inflammatory factors TNF-α and IL-1β, while increase the concentration of anti-inflammatory factor TGF-β [84].

Several studies indicated that exosomes could serve as a novel mechanism to transfer miRNAs between cells to regulate gene expression offering therapeutic effects for liver [85], fibrosis [86], stroke [87], and cardiovascular diseases [88,89]. Moreover, MSC-EVs therapeutic effects have also been described for the adaptive and innate immune response [90]. A variety of studies have examined the immunoregulatory function of MSC-EVs in autoimmune disease models such as autoimmune murine models of type 1 diabetes, experimental autoimmune uveoretinitis, collagen-induced arthritis, synovitis and multiple sclerosis [26]. In this sense, there is an increasing interest to use MSC-EVs in human autoimmune diseases as an alternative to cell therapy. 

Regarding the transfer of mitochondria from MSCs EVs, it was demonstrated that there is an increase of phagocytic activity after mitochondrial transfer from MSC to innate immune cells [79]. For example, the treatment of murine alveolar macrophages with MSC-derived EVs reduce lung injury due to MSCs induce a highly phagocytic and antiinflammatory macrophage phenotype through EV-mediated mitochondrial transfer [91].

Due to MSC-EVs are surrounded by a phospholipid bilayer, they are more stable when freezing and thawing than MSCs, therefore easily prebanked. In addition, EVs present many advantages, including low immunogenicity, lack endogenous tumor-formation potential, in vivo stability, and high delivery efficiency. However, the dose of injected EVs and the route of administration affected their biodistribution pattern [90]. Further studies to better understand the mechanisms underlying MSC-EVs functions are necessary for future EVs-based therapy in the clinic.

## 3. Conclusions

MSC-based cell therapy involves the administration of MSCs as a medicinal product. These cells are characterized by their differentiation potential, migration capacity, immune system modulation, and paracrine activity which can be used as a therapeutic agent in human treatments.

MSC therapy has been investigated extensively in clinical research to evaluate its therapeutic effect in degenerative, immunological, or inflammatory diseases lacking appropriate treatments. First stage results of clinical research have proven safety and tolerability, however, future directions in hMSCs(human MSCs) will involve elucidation of molecular mechanisms by which different microenvironmental cues influence stem cell behavior, followed by translation of these findings to their clinical applications. Paracrine potency and therapeutic efficacy obtained from MSCs are known to vary with source and batch. For successful clinical translation, optimized protocols for MSCs isolation and ex vivo preparation will be necessary. It has been concluded that transplanted MSCs exert their therapeutic effects by acting as “paracrine factor factories” that possess immunomodulation and immune-privileged properties. The high paracrine ability includes EVs release. The use of a cell free preparation comprising of MSC-derived EVs as a conditioning media make these cells an important material for the development of new approaches for non-cell-based therapies. However, there are still new parameters to address in order to determinate a preconditioning treatment to enhance the paracrine potency of MSCs, such as exogenous factors that may help to greatly impact on the MSCs biological properties and eventually on their therapeutic abilities.

## Figures and Tables

**Table 1:** Advantages and disadvantages of mesenchymal stromal cells (MSCs) from the three main sources that have been investigated in clinical studies: bone marrow (BM), adipose tissue (AT), and the umbilical cord (UC).

Source Type	Advantages	Disadvantages
**Adipose Tissue (AT)**	▪ High availability and accessible. ▪ Stem cell isolation of up to 500 times more than BM. ▪ Cells proliferate faster than BM-MSCs (mean doubling time of 40 h). ▪ The immunosuppressive effects of AT-MSCs are stronger than those of BM-MSCs. ▪ Secretion of several angiogenic and antiapoptotic cytokines. ▪ AT-MSCs are more prone to differentiate towards adipocyte lineage.	▪ Inferior osteogenic and chondrogenic potential in comparison to BM-MSCs. ▪ Cell yield and differentiation potential is dependent on donor characteristics (i.e., age).
**Bone Marrow (BM)**	▪ The most extensively investigated. Considered to be the gold standard. ▪ The most common cellular source in clinical trials. Established clinical history. ▪ High chondrogenic and osteogenic potential.	▪ Invasive and painful collection procedure. ▪ Procurement carries the risk of infection. ▪ Limited supply. ▪ Cell yield and differentiation potential is dependent on donor characteristics (i.e., age). ▪ Less proliferative rate in comparison to BM-MSCs and UC-MSCs (mean doubling time of 4 ± 1 days).
**Umbilical Cord (UC)**	▪ Safe and non-invasive collection procedure. ▪ Abundant supply. ▪ UC-MSCs do not age over passages (i.e., senescence). ▪ Hypoimmunogenicity. ▪ Lower risk of graft-versus-host diseases (GvHD). ▪ Higher proliferation potential compared with BM and AT (mean doubling time is 30 h). ▪ Higher expansion and engraftment capacity than BM-MSCs.	▪ UC-MSCs are less effective in inducing osteogenesis compared to BM-MSCs.
